# Multicenter Retrospective Analysis of the Effectiveness and Safety of Rituximab in Korean Patients with Refractory Systemic Lupus Erythematosus

**DOI:** 10.1155/2012/565039

**Published:** 2012-12-09

**Authors:** So-Young Bang, Chang Keun Lee, Young Mo Kang, Hyoun-Ah Kim, Chang-Hee Suh, Won Tae Chung, Yong-Beom Park, Jung-Yoon Choe, Tae-Jong Kim, Yong-Wook Park, Dae-Hyun Yoo, Sang-Cheol Bae, Hye-Soon Lee

**Affiliations:** ^1^Division of Rheumatology, Department of Internal Medicine, Hanyang University Guri Hospital, Gyeonggi-do, Guri-si 471-701, Republic of Korea; ^2^Division of Rheumatology, Department of Internal Medicine, Asan Medical Center, University of Ulsan College of Medicine, Seoul 138-736, Republic of Korea; ^3^Division of Rheumatology, Department of Internal Medicine, Kyungpook National University Hospital, Daegu 700-705, Republic of Korea; ^4^Division of Rheumatology, Department of Internal Medicine, Ajou University Hospital, Gyeonggi-do, Suwon-si 443-721, Republic of Korea; ^5^Division of Rheumatology, Department of Internal Medicine, Dong-A University Hospital, Busan 602-715, Republic of Korea; ^6^Division of Rheumatology, Department of Internal Medicine, Yonsei University College of Medicine, Seoul 120-749, Republic of Korea; ^7^Division of Rheumatology, Department of Internal Medicine, Daegu Catholic University Hospital, Catholic University of Daegu School of Medicine, Daegu 705-718, Republic of Korea; ^8^Division of Rheumatology, Department of Internal Medicine, Chonnam National University Hospital, Gwangju 501-757, Republic of Korea; ^9^Department of Rheumatology, Hanyang University Hospital for Rheumatic Diseases, Seoul 133-792, Republic of Korea

## Abstract

*Objective*. Although two recent randomized placebo-controlled trials of rituximab (RTX) failed to demonstrate efficacy in systemic lupus erythematosus (SLE), clinicians continue to use off-label RTX for cases refractory to current treatments. We evaluated the effectiveness and safety of rituximab for patients with refractory SLE in Korea. *Methods*. We retrospectively analyzed multicenter patients treated with RTX in Korea. *Results*. 39 SLE patients treated with RTX were included in the following manner: lupus nephritis 43.6%, hematologic 33.3%, arthritis 7.8%, myositis 7.8%, and others 7.7%. All patients had responded poorly to at least one conventional immunosuppressive agent (mean 2.5 ± 1.1, cyclophosphamide 43.6%, mycophenolate mofetil 48.7%, and other drugs) before RTX. Clinical improvements (complete or partial remission) occurred in patients with renal disease, hematologic disease, arthritis, myositis, and other manifestations at 6 months after RTX. The SLEDAI score was significantly decreased from 10.8 ± 7.1 at baseline to 6.7 ± 4.0 at 6 months, 6.2 ± 4.1 at 12 months, and 5.5 ± 3.6 at 24 months after RTX (*P* < 0.05). Among 28 clinical responders, 4 patients experienced a relapse of disease at 25 ± 4 months. Infections were noted in 3 patients (7.7%). *Conclusion*. RTX could be an effective and relatively safe therapeutic option in patients with severe refractory SLE until novel B-cell depletion therapy is available.

## 1. Introduction 

Systemic lupus erythematosus (SLE) is associated with diverse clinical manifestations, from skin lesions and arthritis, to damage to vital systems such as the blood and kidney. The management of SLE is based on the type and severity of organ involvement. Patients with SLE are treated with nonsteroidal anti-inflammatory drugs, corticosteroids, antimalarial agents, and immunosuppressive agents including cyclophosphamide, azathioprine, and mycophenolate mofetil, according to their disease activity and the extent of vital organ involvement [1].

B-cell depletion therapy has been shown to be clinically effective in rheumatoid arthritis (RA) [2]. B-cell also have an important role in the pathogenesis of SLE. The regulation of the B-cell receptor is important for specific immune responses to maintain self-tolerance in patients with SLE [3]. B-cell overactivity participates in the activation of the autoimmune processes associated with SLE, such as the production of autoantibodies and various cytokines, and activation of potent antigen-presenting cells [4].

Given these roles of B-cells in SLE pathogenesis, B-cell-targeted therapy has been recently introduced for SLE therapy. The chimeric anti-CD20 antibody, rituximab (RTX) has been reported to be a promising treatment option in several case series and off-label trials in patients with refractory SLE [5–10]. Systematic reviews have also concluded that RTX produces significant improvements in patients with SLE [11, 12]. In contrast, two recent randomized placebo-controlled trials (RCT) of RTX [13, 14] in refractory SLE patients failed to reach their primary end points. Other treatments targeting B cells have shown a beneficial effect in many open-label studies for refractory SLE [5–12]. B-lymphocyte stimulator (BLyS) is associated with the survival of B-cells and immunoglobulin switching. BLyS seems to produce inappropriate survival of B-cells producing various autoantibodies. Belimumab, an anti-BLyS antibody, which is not currently available in Korea, has shown significant clinical efficacy and is approved by the Food and Drug Administration for patients with SLE [15]. 

Here we report a multicenter retrospective study investigating the effectiveness and safety of RTX in Korean patients with refractory SLE.

## 2. Patients and Methods

### 2.1. Patients

Thirty-nine SLE patients refractory to conventional therapies received RTX, who were enrolled from eight tertiary rheumatology clinics in Korea. The patients were all native Koreans and satisfied the American College of Rheumatology 1997 revised criteria for SLE [16]. The study was approved by the local ethics committee in Korea. 

### 2.2. Response Criteria

The clinical and response data for all patients were collected retrospectively by rheumatologists using a uniform format. The responses to treatment were determined by comparing disease activity before RTX and at 3, 6, 12, 24, and 36 months after RTX infusion using response criteria specific to the organ involved. Overall disease activity was assessed using the SLE Disease Activity Index (SLEDAI) [17]. 

For nephritis, complete response was defined as a decrease in proteinuria to <500 mg/day, disappearance of hematuria and cellular casts, and normal estimated glomerular filtration rate (eGFR), and partial response as >50% improvement in renal parameters (proteinuria and eGFR) from pretreatment values [18]. For autoimmune thrombocytopenia, complete response was defined as a platelet count of at least 100 × 10^9^/liter and partial response as a platelet count of 30–100 × 10^9^/liter with more than doubling of the pretreatment count [19]. For autoimmune hemolytic anemia, complete response was defined as a hemoglobin >11 g/dL in women or >12 g/dL in men without hemolysis, and partial response as a hemoglobin level >10 g/dL with more than a 2 g/dL increase from baseline [20]. For arthritis, complete response was defined as resolution of tenderness and swelling of affected joints, and partial response as 50% improvement of those. For other manifestations, complete or partial response was classified based on clinical symptoms, SLEDAI score, laboratory data, and physical examination by rheumatologists. Those who failed to meet the above criteria for partial or complete response were defined as nonresponders.

### 2.3. RTX Dose

Dosing of RTX differed depending on the decision of rheumatologists or disease severity. Twenty-three (59.0%) patients received two 500 mg doses of RTX 2 weeks apart, five (12.8%) received 375 mg/m^2^/week for 4 weeks, and four (10.3%) received two 1000 mg doses of RTX 2 weeks apart (Table 1).

### 2.4. Statistical Analysis

The Chi-square test was used to compare responders and nonresponders. Differences in continuous variables were analyzed using the Mann-Whitney *U* test or *t*-test. All statistical analyses were performed with PASW Statistics 18 for Windows (IBM SPSS, USA).

## 3. Results

### 3.1. Study Population

Thirty-nine patients with refractory SLE treated with RTX were included from 8 centers in Korea and did not have previously received RTX (Table 1). The principle manifestations for which RTX administration was started, were lupus nephritis (*n* = 17), hematologic disorder (*n* = 13), arthritis (*n* = 3), myositis (*n* = 3), vasculitis (*n* = 2), and enteritis (*n* = 1). The mean duration of disease was 4.7 ± 3.5 years. *Refractory SLE was defined as patients who failed to meet at least partial response of the each criteria after over 3 months of continuous conventional treatment prior to rituximab*.

In patients with lupus nephritis, mean proteinuria was 5.1 ± 2.9 g/day (mean ± SD) at initial RTX infusion, and in 15 of 17 patients, the diagnosis was confirmed by kidney biopsy. Lupus nephritis was classified as active class III (*n* = 2), class IV (*n* = 7), class V (*n* = 1), class III + V (*n* = 4), and class IV + V (*n* = 1) by the International Society of Nephrology/Renal Pathology Society 2003 criteria [21]. Hematologic disorders included autoimmune thrombocytopenia (*n* = 11) and hemolytic anemia (*n* = 2). In patients with autoimmune thrombocytopenia, the mean platelet count was 43 ± 5.3 × 10^9^/liter (mean ± SD). In patients with hemolytic anemia, the mean hemoglobin level was 6.7 ± 0.9 g/dL (mean ± SD). The SLEDAI score was 10.8 ± 7.1 at pretreatment, including mild disease activity (score < 6) in 10 patients (26%), moderate activity (score 6–10) in 13 patients (33%), high activity (score 11–19) in 10 patients (26%), and very high activity (score > 19) in 6 patients (15%). 

### 3.2. Treatment with RTX and Immunosuppressive Agents

Previous conventional treatment failure was defined as at least one course (mean ± SD, 2.5 ± 1.1) of standard immunosuppressive agents (azathioprine, mycophenolate mofetil, cyclophosphamide, cyclosporine, intravenous (IV) Immunoglobulin, methotrexate, tacrolimus, and others), in combination with corticosteroids (100%) and/or hydroxychloroquine (84.6%). As shown in Table 1, the immunosuppressive agents administered before RTX included mycophenolate mofetil in 48.7% (*n* = 19), cyclophosphamide in 43.6% (*n* = 17), azathioprine in 33.3% (*n* = 13), cyclosporine in 23.1% (*n* = 9), IV immunoglobulin in 17.9% (*n* = 7), methotrexate in 7.7% (*n* = 3), tacrolimus in 2.6% (*n* = 1), TNF blockade in 2.6% (*n* = 1), and others in 5.1% (*n* = 2) of patients. All patients with lupus nephritis (*n* = 17) had previously received cyclophosphamide or mycophenolate mofetil. Nephritis was refractory to cyclophosphamide in 13 patients (76%), to mycophenolate mofetil in 11 patients (65%), and to both drugs in 7 patients (41%). All patients with autoimmune thrombocytopenia (*n* = 11) were refractory to at least one conventional immunosuppressive treatment. Autoimmune thrombocytopenia was refractory to IV immunoglobulins in 64% (*n* = 7), to azathioprine in 45% (*n* = 5), to mycophenolate mofetil in 27% (*n* = 3), to cyclophosphamide in 27% (*n* = 3), and to cyclosporine in 27% (*n* = 3) patients.

Concomitant immunosuppressive agents used with RTX are summarized in Table 1. Glucocorticoids were administered with RTX in 87.2% of cases (*n* = 34), with a prednisone dosage of 32.4 ± 21.7 mg/day (mean ± SD). Hydroxychloroquine and azathioprine were used with RTX in 84.6% (*n* = 33) and 59.0% (*n* = 23) of cases, respectively. The mean number of concomitant immunosuppressive agents was 2.1 ± 0.9 (mean ± SD).

### 3.3. Effectiveness of RTX

Data on the 37 patients with 6-month followup or more were analyzed for effectiveness of RTX. 

**Figure 1:**
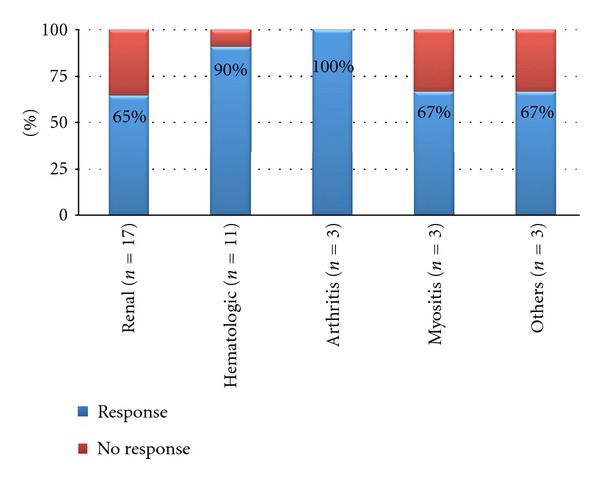
Response at 6 months after RTX treatment in patients with refractory SLE.

Complete or partial response to RTX after 6 months was found in 28 (75.6%) patients: 11 (64.7%) lupus nephritis (0 complete and 11 partial response), 10 (90.1%) hematologic disorder (3 complete and 7 partial response), 3 (100%) arthritis (0 complete and 3 partial response), 2 (66.7%) myositis (0 complete and 2 partial response), and 2 (66.7%) others (0 complete and 2 partial response) (see Figure 1). Responders and nonresponders at 6 months after RTX did not differ significantly according to age, sex, disease duration, or RTX dose (Table 2).

**Figure 2:**
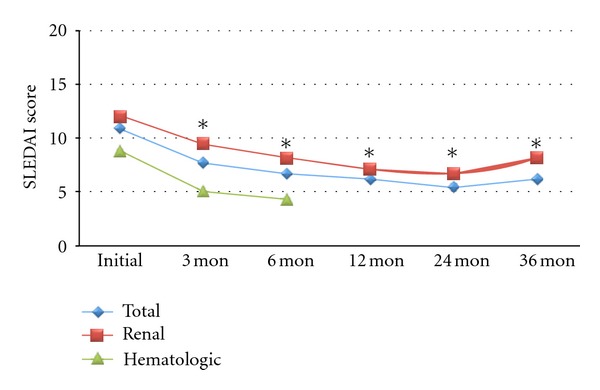
SLEDAI score after RTX treatment in patients with refractory SLE. *P value <0.05.

The total SLEDAI score was decreased from 10.8 ± 7.1 at baseline to 6.7 ± 4.0 at 6 months (24.4% change, *P* < 0.001), 6.2 ± 4.1 at 12 months (14.8% change, *P* = 0.007), 5.5 ± 3.6 at 24 months (35.8% change, *P* = 0.003), and 6.2 ± 4.3 at 36 months (37.3% change, *P* = 0.009) after RTX (Figure 2). The SLEDAI score of patients with lupus nephritis (*n* = 17) was decreased from 11.9 ± 5.1 at baseline to 8.1 ± 3.6 at 6 months (24.4% change, *P* = 0.009), 7.1 ± 4.4 at 12 months (28.8% change, *P* = 0.027), 6.7 ± 3.5 at 18 months (33.3% change, *P* = 0.014), 8.1 ± 3.5 at 24 months (29.1% change, *P* = 0.024), and 6.8 ± 3.5 at 36 months (*P* = 0.027). The SLEDAI score of patients with hematologic disorders (*n* = 13) was decreased from 8.8 ± 7.5 at baseline to 4.4 ± 4.1 at 6 months (29.7% change, *P* = 0.012) after RTX.

Among the 28 clinical responders after the first course of RTX, 4 patients (1 lupus nephritis, 3 thrombocytopenia) experienced a relapse of disease at 25 ± 4 months. After readministration with RTX, all these relapsed patients had responded at 6 months (1 complete, 3 partial response).

### 3.4. Safety of RTX

Adverse events were observed in 7 of 39 patients receiving RTX (17.9%). Four patients experienced mild infusion reactions (rash or myalgia) (10.3%), but severe infusion reactions such as anaphylactic shock or hypotension were not noted (Table 3).

Infections were observed in 3 patients (7.7%) who developed pneumonia, tuberculosis, and an abscess after RTX therapy. Among them, one patient died of septic shock due to a multifocal abscess in the muscles of the hip.

## 4. Discussion

In the present multicenter study, RTX was effective in 75.6% of patients (28/37) with refractory SLE who had responded poorly to conventional immunosuppressive treatment. The SLEDAI score was significantly lower after RTX than at baseline and remained so after 3 years. RTX infusion was relatively well tolerated in this study, with only 17.9% of patients experiencing adverse events. These findings are in keeping with those of previous open-label studies of RTX in SLE [5–12]. 

Two RCTs of RTX in SLE patients without lupus nephritis (Exploratory Phase II/III SLE Evaluation of Rituximab [EXPLORER]) [13] and with lupus nephritis (Lupus Nephritis Assessment with Rituximab [LUNAR]) [14] have failed to demonstrate efficacy of RTX over placebo.

The EXPLORER trial [13] did not meet its primary end point comparing RTX to placebo. A total of 257 patients were enrolled from multiple centers in the USA and were excluded if they had lupus nephritis, severe central nervous system involvement or were currently being treated with an immunosuppressive drug. Differences in regard to the primary end point between the two groups were not observed. However, a partial clinical response was reported in the RTX group (20%) compared to placebo (6.3%) on subgroup analysis. The major differences between the EXPLORER trial and our study are the ethnicity of the patients (mainly Caucasian versus all Korean), and the fact that patients with lupus nephritis were excluded from the RCT whereas we included some with moderate to severe lupus nephritis.

In the LUNAR trial [14], patients with proliferative (class III or IV) lupus nephritis were randomized to RTX versus placebo and all treated with high-dose glucocorticoids and mycophenolate mofetil. However, the primary end point for RTX was not met, although RTX improved serologic markers (anti-dsDNA/complement) and responders at week 52 showed a greater depletion of CD19 cells compared with nonresponders. 

The optimal dosing regimen for RTX in SLE remains unclear. In RCT studies, RTX 1,000 mg × 2 infusions were used and responders had a lower B-cell counts. In the present study, responders received a higher RTX dose (1285 ± 460 mg) than nonresponders (1062 ± 417 mg), but the difference was not statistically significant. A trial in RA patients comparing dosing regimens using 500 mg and 1000 mg doses found that clinical response correlated with absolute preplasma cell count following RTX treatment rather than RTX dose per se [22]. The same investigators have also used highly sensitive flow cytometric analysis of B-cell subsets to confirm this observation [23]. It is possible therefore those variations in baseline B-cell counts account for the variability in clinical response to RTX observed in the SLE patients in our study. The qualitative properties of the B-cell subtypes may be important markers of treatment response in SLE.

It is important to note that our study differs from the LUNAR trial in that all patients with lupus nephritis included in our study were refractory to cyclophosphamide and/or mycophenolate mofetil. Two trials [13, 14] included patients who received high-dose corticosteroids and immunosuppressive drugs in both the RTX and placebo groups and excluded patients previously treated with cyclophosphamide. However, many open-label studies [5–12, 24, 25] have yielded favorable results for RTX in refractory SLE. Prospective data from the French AutoImmunity and Rituximab (AIR) registry [9] showed safety and clinical efficacy of RTX, reporting articular, renal, and hematologic improvements (72%, 74%, and 88%, resp.) including in patients with refractory SLE. In addition, patients with lupus nephritis of European cohorts were showed 67% improvements at 12 months after RTX therapy [25], suggesting that RTX has therapeutic biological effects in SLE. 

The limitations of our study include its relatively small population size, retrospective and observational design, and missing immunological data such as B-cell counts (CD19, CD20) or RTX human antichimeric antibody. In addition, the SLEDAI scores do not record improving or worsening, and may be not sensitive to small changes of disease activity after treatment. Further studies are needed to confirm RTX dose and concomitant immunosuppressive therapy.

In summary, RTX might not be recommendable as first-line treatment for patients with SLE who have potential to respond well to the conventional treatment. However, many studies including the present Korean multicenter retrospective study supported that RTX could be effective and relatively safe therapeutic option in patients with severe refractory SLE until new novel B-cell depletion therapy is widely available. Further randomized controlled trials in patients with refractory SLE are needed.

## Figures and Tables

**Table 1:** Characteristics of the 39 SLE patients receiving rituximab.

Age, mean ± SD years	32.1 ± 8.6
Female, %	92.1
Disease duration, mean ± SD years	4.7 ± 3.5
Major organ involved, % (*n*)	
Nephritis	43.6 (17)
Hematologic	33.3 (13)
Arthritis	7.8 (3)
Myositis	7.8 (3)
Vasculitis	5.1 (2)
Enteritis	2.6 (1)
Disease activity before rituximab	
SLEDAI score, mean ± SD years	10.8 ± 7.1
Anti-DNA antibody, % (*n*)	48.7 (19)
C3, mean ± SD g/dL	70.9 ± 27.9
C4, mean ± SD g/dL	15.6 ± 12.6
Rituximab administration, % (*n*)	
500 mg × 2 infusions	59.0 (23)
1000 mg × 2 infusions	10.3 (4)
375 mg/m^2^ × 4 infusions	12.8 (5)
500 mg × 1 infusion	7.7 (3)
Other regimen	10.2 (4)

Previous immunosuppressive agents, % (*n*)	
Mycophenolate mofetil	48.7 (19)
Cyclophosphamide	43.6 (17)
Azathioprine	33.3 (13)
Cyclosporine	23.1 (9)
IV immunoglobulin	17.9 (7)
Methotrexate	7.7 (3)
Tacrolimus	2.6 (1)
TNF blocker	2.6 (1)
Others	5.1 (2)
Number of immunosuppressive agents, mean ± SD	2.5 ± 1.1
Rituximab-concomitant immunosuppressive agents, % (*n*)	
Glucocorticoids	87.2 (34)
Dosage, mean ± SD mg/day	32.4 ± 21.7
Hydroxychloroquine	84.6 (33)
Azathioprine	59.0 (23)
Cyclosporine	28.2 (11)
Mycophenolate mofetil	23.1 (9)
Cyclophosphamide	2.6 (1)
Number of immunosuppressive agents, mean ± SD	2.1 ± 0.9

*Values are % (number) of patients. SLEDAI: systemic lupus erythematosus disease activity index.

**Table 2:** Comparison of responders and nonresponders at 6 months after rituximab administration (*n* = 37).

	Nonresponders (*n* = 9)	Responders (*n* = 28)	*P*
Age, mean ± SD	36.6 ± 8.8	30.8 ± 8.7	0.11
Female, %	77.8	96.3	0.06
Disease duration, mean ± SD	4.1 ± 3.1	4.9 ± 3.6	0.61
Rituximab dose, mean ± SD	1062 ± 417	1285 ± 460	0.22

**Table 3:** Adverse events in 39 SLE patients receiving rituximab.

Total adverse events, % (*n*)	17.9 (7)
Infusion reaction (rash, fever, myalgia)	10.3 (4)
Infection (pneumonia, tuberculosis, abscess)	7.7 (3)
Death due to severe infection, % (*n*)	2.7 (1)

*Values are % (number) of patients.
